# Mesenchymal Stem Cells in Immune-Mediated Bone Marrow Failure Syndromes

**DOI:** 10.1155/2013/265608

**Published:** 2013-12-10

**Authors:** Maria-Christina Kastrinaki, Konstantia Pavlaki, Aristea K. Batsali, Elisavet Kouvidi, Irene Mavroudi, Charalampos Pontikoglou, Helen A. Papadaki

**Affiliations:** Department of Haematology, University of Crete School of Medicine, 70013 Heraklion, Crete, Greece

## Abstract

Immune-mediated bone marrow failure syndromes (BMFS) are characterized by ineffective marrow haemopoiesis and subsequent peripheral cytopenias. Ineffective haemopoiesis is the result of a complex marrow deregulation including genetic, epigenetic, and immune-mediated alterations in haemopoietic stem/progenitor cells, as well as abnormal haemopoietic-to-stromal cell interactions, with abnormal release of haemopoietic growth factors, chemokines, and inhibitors. Mesenchymal stem/stromal cells (MSCs) and their progeny (i.e., osteoblasts, adipocytes, and reticular cells) are considered as key cellular components of the bone marrow haemopoietic niche. MSCs may interfere with haemopoietic as well as immune regulation. Evidence suggests that bone marrow MSCs may be involved in immune-mediated BMFS underlying pathophysiology, harboring either native abnormalities and/or secondary defects, caused by exposure to activated marrow components. This review summarizes previous as well as more recent information related to the biologic/functional characteristics of bone marrow MSCs in myelodysplastic syndromes, acquired aplastic anemia, and chronic idiopathic neutropenia.

## 1. Introduction

Immune-mediated bone marrow failure syndromes (BMFS), such as the myelodysplastic syndromes, acquired aplastic anemia, or chronic idiopathic neutropenia, are characterized by ineffective marrow haemopoiesis and subsequent peripheral cytopenias. Pathogenetic mechanisms involve a complex marrow deregulation, including genetic and epigenetic alterations, resulting in aberrant release of haemopoietic growth factors and inhibitors in the marrow, deregulated immune manifestations, all resulting in defective haemopoietic maturation and increased haemopoietic cell apoptosis.

Normal haemopoiesis is regulated in the marrow by an extended network of specialized niches, maintaining haemopoietic stem cell (HSC) self-renewal and orchestrating HSC proliferation and differentiation to all blood cell types. Key cellular components of the bone marrow (BM) haemopoietic microenvironment include osteoblasts, sinusoidal endothelial cells, macrophages, adipocytes, and reticular cells, orchestrating the maintenance, proliferation, and differentiation of haemopoietic stem and progenitor cells (HSPCs). Osteoblasts, adipocytes, and reticular cells of the marrow stroma derive from a common progenitor cell, the mesenchymal stem/stromal cell (MSC) [1–5]. Since MSCs and their progeny are among the main components of the marrow stroma, it is reasonable to assume that patient BM MSCs may be partially defective, harboring either native abnormalities and/or secondary defects, due to the long-term exposure to activated marrow components. MSCs could be involved in various pathogenetic mechanisms. MSC haemopoietic supportive capacity, in terms of production of haemopoietic growth factors, or inhibitors, or generation of extracellular matrix, may be defective. MSC differentiation capacity could also indirectly influence haemopoiesis, by controlling marrow cell composition: osteoblasts favor haemopoiesis, yet adipocytes inhibit haemopoiesis. Furthermore MSC immune functions may be deregulated, contributing to the establishment or persistence of the immune-mediated disease manifestations.

The purpose of this review is to summarize and discuss literature information regarding the biologic and functional characteristics of BM MSCs in the immune-mediated BMFS, namely, myelodysplastic syndromes, chronic idiopathic neutropenia, and aplastic anemia.

## 2. BM MSC Properties

Mesenchymal stem/stromal Cells (MSCs) are multipotent progenitors able to differentiate into the mesenchymal cell types of adipocytes, chondrocytes, and osteoblasts, additionally showing a wider potency able to differentiate to other cell types, such as myocytes, hepatocytes, or even neurons [3, 6–8]. Originally isolated from the bone marrow [9], MSCs have also been isolated from a variety of other tissues, including dental pulp, bone, lung, adipose tissue, and umbilical cord [10–13]. MSCs have drawn much attention during the last decade in the field of regenerative medicine, mainly due to their capacity to differentiate into specific cell types, their abundant production of soluble growth factors and cytokines, and their immunomodulating properties.

As proposed by the International Society for Cellular Therapy three criteria are used to define MSCs: adherence to plastic, specific surface antigen expression, and multipotent differentiation potential (the latter is being tested by cytochemical stains and evaluation of specific gene expression) [14]. Regarding cell immunophenotype, MSCs are positive for CD73, CD90, and CD105 among numerous other cell surface antigens, while being negative for haemopoietic cell markers (such as CD14, CD34, and CD45), class II major histocompatibility complex (HLA-DR), or costimulatory molecules (CD80, CD86) [14]. Due to the absent/low expression of MHC class II molecules, MSCs are immunoprivileged cells and have been used in allo- as well as xenotransplantations. Native BM MSCs are somewhat immunophenotypically different from in vitro expanded cells. Since there is no unique MSC marker, several different cell markers have been used to follow native BM MSCs, such as SSEA4, LNGFR (CD271), or CXCL12 (SDF-1) [15–17].

Evidence suggests that BM MSCs and their progeny are important haemopoietic regulators: osteoprogenitors, osteoblasts, adipocytes, and reticular perivascular cells are all key components of the hematopoietic niche [17–19]. The endosteum, comprising of different types of osteolineage cells, plays a critical role in the maintenance and homing of HSCs. Osteocytes and their role are under investigation. For instance, the CD45−/Ter119−/OPN+ osteoblasts were shown to rapidly expand in vivo, following cyclophosphamide/G-CSF treatment, correlating to HSC proliferation and mobilization, and treated isolated OPN+ cells improved their in vitro haemopoietic supportive ability [20]. The maturation state of osteoblasts appears to be related to the haemopoietic supportive functions, with immature osteoblasts being more efficient in HSC support [21]. Adipocytes on the other hand inhibit haemopoiesis, with increased levels of BM adipogenesis inversely correlating to HSC numbers [22].

In vivo BM MSCs have been described in close proximity to HSCs, as perivascular CXCL12 abundant reticular (CARs) cells [17, 23, 24] and Nestin+/CD45− cells [25]. The importance of CXCL12-CXCR4 signaling in maintenance and homing of both HSCs and immune cells is well established [26–29]. CAR cells were characterized as a sparse cell population forming a network within the bone marrow, in close contact with HSCs and immune cells, surrounding sinusoidal endothelial cells. Selective CAR cell depletion negatively affects HSC size, number, and proliferation [23]. Moreover, CAR cells can differentiate in vitro to adipocytes and osteoblasts, strongly suggesting they are MSCs. Regarding the perivascular Nestin+/CD45− cells, these were also shown to differentiate to adipocytes and osteoblasts, though with a very low efficiency, and rather seem to comprise of a more heterogeneous cell population, containing mostly endothelial cells and possibly CAR cells as well [25, 30].

Cultured BM MSCs produce many soluble factors known to be important for HSC maintenance (Table 1). More particularly, they produce stem cell factor (SCF), Flt3 ligand, thrombopoietin, leukemia inhibiting factor (LIF), interleukin (IL)-6, IL-7, IL-8, IL-11, IL-12, IL-14, IL-15, GM-CSF, and M-CSF [4, 31, 32]. Cultured BM MSCs also secrete a wide range of chemokines, namely, CCL2, CCL3, CCL4, CCL5, CCL7, CCL20, CCL26, CXCL1, CXCL2, CXCL5, CXCL8, CXCL10, CXCL11, and CXCL12 [33]. Additionally, MSCs may also interact with their close environment via adhesion molecules, such as ICAM-1 and VCAM-1 (CD106), and by producing extracellular matrix proteins (such as fibronectin, integrins, and collagen).

Moreover, MSCs can elicit immunosuppressive effects in vitro and in vivo. The most striking example of MSC-immunosuppression is described by their beneficial effects in patients with acute graft versus host disease [34]. MSCs can interact/inhibit both innate and adaptive immunity cells (Table 2): they can inhibit in vitro cell proliferation and function of T cells [35], B cells [36, 37], natural killer (NK), and dendritic cells (DC) [38] or promote regulatory T lymphocytes (Tregs) [39]. MSCs exert immunomodulation through prostaglandin E2 (PGE2), TGF-*β*, IL-6, IL-10, human leukocyte antigen-G5 (HLA-G5), matrix metalloproteinases, indoleamine-2,3-dioxygenase (IDO), and nitric oxide [40–43]. It is now apparent that MSCs are not constitutively immunosuppressive and instead require a “licensing” step, provided by inflammatory molecules, like IFN*γ* and TNF-*α*, or toll-like receptor (TLR) ligands [44]. It has been suggested that MSC functional characteristics depend on and are modified by environmental cues: for example, depending on environmental IFN*γ* concentration, MSCs can be modified to act either as antigen-presenting cells or can be immunosuppressive [45, 46].

As the BMFS marrow is characterized by abnormalities, for instance, deranged expression of cytokines or chemokines, such as IFN*γ*, TNF-*α*, IL-1*β*, or IL-6, affecting various cellular marrow components, patient MSCs may also be affected and their immune or other properties could be modified accordingly [47]. Besides the possibility of a genuine MSC defect, cannot be ruled out. Next we summarize earlier literature and present latest data regarding BM MSCs of immune-mediated BMFSs.

## 3. Myelodysplastic Syndromes

Myelodysplastic syndromes (MDS) are heterogeneous clonal HSC disorders, characterized by ineffective haemopoiesis, with varying degrees of peripheral cytopenias and increased risk of transformation to acute myeloid leukemia (AML). MDS patient HSCs acquire multiple genetic and epigenetic abnormalities, progressively leading to disease evolution. Depending on disease manifestations and evolution, MDS are classified to several subtypes [48, 49]. Early (low-risk) stages of MDS are characterized by ineffective HSC maturation, combined with increased apoptosis of haemopoietic progenitors, leading to peripheral cytopenias. More advanced (high-risk) stages of MDS on the other hand are characterized by apoptosis resistance, increased proliferation, and final transformation to AML [50–54]. Immune-mediated pathogenesis in MDS is suggested by the fact that a subset of patients responds to immunosuppressive therapy with antithymocyte globulin (ATG), as well as by several abnormal immune manifestations of the disease, including T cell mediated inhibition of haemopoiesis, irregularities of the TCR repertoire, decreased and functionally impaired regulatory T cells (Tregs) in low-risk MDS marrow, and increased Treg number and activity in high-risk MDS [55].

Although MDS is considered as a clonal haemopoietic progenitor disease, there is accumulating evidence to suggest that the bone marrow microenvironment also contributes to disease pathology [56, 57]. For example, animal studies show that marrow microenvironmental deregulations are sufficient to cause MDS [58, 59].

When it comes to BM MSC involvement in MDS, published data have been rather inconsistent and contradictory. This is mostly attributed to the variability of methodologies used for the isolation and in vitro expansion of BM MSCs, as well as to MDS patient heterogeneity, confusing result interpretation. It is now obvious that MDS pathophysiology varies depending on disease subtype and stage, with a clear discrimination between low-risk and high-risk MDS stages, displaying different biologic characteristics, including different immune manifestations. Some of the described studies below have been conducted with separate MDS subtypes, while others were conducted by pooling all patient data.

Many studies have tried to investigate the possibility of an underlying defect in patient-derived MSCs. Since BM MSCs represent a rare marrow population, the majority of studies were conducted in culture-expanded cells. With regard to the general MSC characteristics, we have found in accordance with others that patient-derived MSCs have normal immunophenotype [60–62], although some abnormalities in the immunophenotype with respect to CD90, CD104, and CD105 expression have been described [63–65]. More recent studies also support the normal immunophenotype for culture-expanded cells [66–69]. Nevertheless, patient MSCs appear frequently disorganized in both size and shape.

The majority of studies reveal defective growth characteristics for culture-expanded MDS-derived MSCs compared to normal cells [62, 65, 66, 70, 71], although normal growth has also been reported [67, 72]. The reported growth deficiencies for patient MSCs include slower proliferation rate, fewer overall population doublings, less clonogenicity (CFU-F), and increased senescence (SA-b-gal). The described growth deficiencies are not attributed to increased apoptosis [65], and literature data, although somehow controversial, are rather suggestive of premature senescence. Recently in a large study conducted by Geyh and coworkers, which included 121 subjects representing all the major MDS subtypes, growth deficiencies and significantly increased senescence were found for all MDS-derived MSC cultures [66]. As premature telomere length shortening is related to replicative exhaustion and replicative senescence, we estimated telomere length and did not find any premature telomere shortening in MDS-derived MSCs (unpublished data), in accordance with previously reported data for marrow stroma in MDS [73]. Aiming to investigate further the underlying cause of defective cell growth, we have recently found that patient MSCs display a significant upregulation of the noncanonical WNT expression and downregulation of canonical WNT, along with upregulation of canonical WNT inhibitors (unpublished data). The multifunctional WNT pathway signaling, implicated in controlling MSC proliferation and differentiation, as well as haemopoietic regulation, needs further exploration.

MSCs are defined as multipotent stromal cells. The differentiation capacity to adipocytes, osteoblasts, and chondrocytes of MDS-derived MSCs has also been investigated, since MSC multipotency may indirectly influence the marrow haemopoietic capacity, by regulating particular cell balances in the marrow. In accordance with other studies, we have found the differentiation capacity of patient MSCs within the normal limits [61, 62, 66, 67], while others have reported impaired differentiation capacity [65, 66, 70]. We recently found that patient MSCs under basic (nondifferentiating) conditions displayed decreased expression of adipogenesis and osteogenesis-related genes (unpublished data), suggesting defective MSC-lineage priming [74]. Inconsistencies in the reported literature could be attributed to the different methodologies (nonquantitative cytochemical stains versus quantitative RT-PCR) or to patient heterogeneity or even to inadequate number of patients. When large numbers of different MDS subtypes were assessed, defective osteogenic potential was demonstrated for all MDS samples by cytochemical stains and reduced expression of Osterix, and Osteocalcin [66]. The altered methylation signature of MDS-derived MSCs further supported a deregulated osteogenic potential, showing a strong hypermethylation of the TBX15 transcription factor, necessary for normal murine bone formation, to all patients, along with decreased TBX15 gene expression [66].

In a large percentage of MDS patients, multiple genetic abnormalities are detected in marrow haemopoietic cells. Patient HSCs show genetic instability, progressively acquiring multiple genetic mutations. Many studies have been conducted to unravel whether MDS-derived MSCs are devoid of, or harbor the same, or possibly other genetic aberrations. If MDS-derived MSCs bear the genetic abnormalities of patient HSCs, it would be suggestive that both patient MSCs and HSCs derive from the same clone. Moreover, given the haemopoietic cells' genetic instability in MDS, it is of interest to explore whether this is also reflected upon patient MSCs. Published data show that, although patient MSCs are devoid of the typical chromosomal abnormalities detected in haemopoietic cells [75, 76], they frequently bear various genetic aberrations [60–62, 65, 77, 78]. The frequency of patient MSCs being found with genetic abnormalities varies from study to study, probably due to the different sensitivities of the several techniques used by investigators: from the classic G-banding karyotype and FISH to the most sophisticated and sensitive array-CGH. Typically, when the latter was used, genetic alterations were found to all patients [65]. Findings are rather suggestive of a BM genetic unstable background in MDS, either inherent or established by the long-term exposure to marrow activated environment, where MSC genetic instability could further contribute to disease pathogenesis. Nevertheless, the diverse genetic aberrations described for patient MSCs are of unknown biologic significance so far.

Cellular epigenetic modifications are of great importance in gene expression and therefore cell function. Recently, epigenetic changes were described, in terms of altered methylation profile, for refractory anemia with excess blasts (RAEB) MDS and refractory cytopenia with multilineage dysplasia (RCMD) MDS MSCs compared to normal MSCs [66], suggesting altered cell function for MDS-derived MSCs.

Within the context of immune-mediated pathogenesis, MDS-derived MSC immune properties were investigated. Although we have found that culture-expanded patient MSCs can efficiently inhibit T cell mitogen-induced proliferation in vitro [62], the majority of studies are suggestive of impaired immune properties [67, 72, 79–81]. Our results differ from others', most probably because our patients included both low-risk and high-risk MDS. Lately the immunoregulatory functions of low-risk and high-risk MDS were investigated [72, 80] and, interestingly, MSCs from low-risk MDS (but not from high-risk) displayed impaired immunosuppressive functions, but when the same data were analyzed including both low- and high-risk patients, the immune properties of MSCs did not differ from normal. Low-risk MDS-derived MSCs manifested ineffective inhibition of mitogen-induced T cell proliferation, as well as ineffective induction of Tregs. Moreover, MSCs from low-risk MDS patients (but not high-risk) could not effectively inhibit DC maturation. Lastly impaired MSC immunosuppression was reported for refractory anemia (RA) MDS patients [79, 81]. These data are suggestive of impaired immune functions for MDS-derived MSCs, although not necessarily attributed to all MDS subtypes.

Regarding the in vitro haemopoietic supporting capacity of BM MSCs in MDS, some authors propose that culture-expanded MSCs are able to support in vitro growth of haemopoietic progenitors [61, 76], while others have reported contradictory results (including our unpublished data) [66, 67, 70]. Published data are confusing, most probably due to differences in methodology: some have used autologous CD34+ cells, while others used normal heterologous or umbilical cord CD34+ cells. Regarding molecules that are implicated with MDS, we have shown that the proinflammatory cytokines TNF-*α*, IL-1*β*, IL-6 and the growth-promoting cytokines VEGF and SDF-1, frequently over-expressed in patient marrow, were normal in culture supernatants [62], although IL-6 and IL-1*β* were found increased by others [61, 67]. Recently altered expression of molecules involved in haemopoietic regulation was reported, including the haemopoietic inhibitor osteopontin (OPN), kit ligand, angiopoietin-1, and Jagged1, as well as altered expression of various immune regulating chemokines [66]. Patient heterogeneity could account for literature discrepancies. For instance, TGF-*β* was underexpressed only in low-risk MDS MSCs, while HGF was overexpressed only in high-risk MSCs, further demonstrating MSC differential functions between low-risk and high-risk patients [72]. Focal adhesion molecules, through which MSCs interact with their environment, may also affect MSC-HSC interactions, as suggested by focal adhesion irregularities in MDS-derived MSCs, that negatively impacted patient haemopoietic precursors' clonogenicity [68]. Focal adhesion deregulations involved protein subcellular localization, rather than an irregular overall expression.

While most of the aforementioned reports have been carried out on culture-expanded MSCs, recently Flores-Figueroa and coworkers have used immunohistochemistry and immunofluorescence in MDS/AML and benign marrow biopsies, thereby showing a significant expansion of the BM MSC network in MDS [30]. Provided data reveal that in benign marrow CXCL12+/CD271+/ALP+ MSCs are forming an arborizing cell network encircling vasculature, coating bony trabeculae and adipocytes, and spreading out through the parenchyma. In benign marrow the chemokine CXCL12 is predominantly expressed by the vascular endothelium and to a lower level by MSCs. In contrast, in MDS marrow (but not AML), the parenchymal CXCL12+/ALP+ MSC population was significantly expanded and additionally overexpressed CXCL12, providing a more widespread and increased CXCL12 expression. The vast majority of CD34+ HSPCs in both benign and MDS marrow were found in intimate contact with CD271+ MSCs, with a preferred perivascular distribution, in close contact with perivascular MSCs. Researchers suggested a novel model of how MDS stroma affects haemopoiesis, where expansion of CXCL12+ expressing MSCs in MDS stroma may expose adjacent CD34+ HSPCs to increased contact-mediated signaling with CXCL12-expressing cells, probably providing CD34+ HSPCs with abnormal survival, proliferation, or homing signaling [26–30].

Perhaps the most striking evidence that BM MSCs may play an important role in the induction of MDS or leukemia comes from a study in mice, where selective deletion in osteoprogenitors of Dicer1, a RNaseIII endonuclease, essential for miRNA biogenesis and RNA processing, resulted in development of myelodysplasia and secondary leukemia [58]. Dicer1 was not deleted in HSCs/HPCs, interfering only with osteoprogenitors, and quite interestingly Dicer1 was also found downregulated in MDS-derived MSCs [82]. The induction of MDS and leukemia by Dicer1 selective deletion is not proving that MDS is caused by deranged MSCs but is rather suggestive that bone marrow MSC deregulations are sufficient to induce MDS. The normal reconstitution of the host bone marrow, after allogeneic haemopoietic cell transplantation, is suggestive of normal overall stroma in MDS. Nevertheless, patient MSCs bear genetic and epigenetic aberrations, suffer from growth deficiencies, and frequently show impaired immune and haemopoietic support functions, raising the possibility that all these MSC derangements could result and may be imposed by the long-term exposure of patient MSCs to an abnormal marrow environment.

## 4. Aplastic Anemia

Acquired aplastic anemia (AA) is characterized by aplastic or hypoplastic, fatty marrow, with peripheral pancytopenia of varying degree [83]. In some patients, AA is associated with chemical or drug exposure, or viral infections, but the underlying etiology remains elusive. The marrow is severely damaged during disease evolution, with reduced BM capacity, excessive adipocytes, reduced capillaries, and increased apoptosis of HSPCs. Strong evidence of the immune-mediated pathogenesis in acquired AA comes from the success of immunosuppressive therapies in treating AA and is also suggested by several immune manifestations, including aberrations in immune cell numbers and functions, as well as aberrations in various immune molecules (reviewed in [84]). More specifically, Th1 lymphocytes [85], cytotoxic CD8+ T cells [86], and DCs [87] are significantly increased in patients, while regulatory Tregs are decreased [88]. Moreover, a variety of immune molecules are abnormally expressed in patient marrow and periphery. The major mediators of the haemopoietic suppression in AA are IFN*γ* and TNF-*α*, both excessively produced in patients [89–92]. Other aberrantly expressed immune molecules include IL-2, IL-6, IL-8, IL-12, IL-17, and MIP-1*α* [84].

Several investigators have shown that in acquired AA patients CD34+ haemopoietic progenitors are reduced in number [93–95]. Moreover, HSPC apoptosis is significantly increased in patients [96, 97]. In vitro data suggest that patient self-reactive Th1 cells [98, 99] and cytotoxic CD8+ T cells [100] induce HSPC apoptosis, via the overexpressed Fas/FasL pathway [101, 102] or through IFN*γ*/TNF-*α* [89–92]. Moreover, patient aberrant cytokine/chemokine production may further contribute to HSPC destruction.

BM MSCs from AA patients have the typical MSC immunophenotype, although once again aberrant cell morphology has been described [103–105], as well as proliferative defects, lower clonogenicity and increased cell apoptosis [105–107]. Moreover the differentiation capacity of AA-derived BM MSCs appears defective [105–107]. Trying to explain the fatty marrow in patients, it was suggested that AA-derived MSCs overexpress the key adipogenic regulator PPARG, possibly due to a significant decrease of the negative regulator GATA-2 [104].

Given the context of immune-mediated pathogenesis in AA, it is of particular interest to explore BM MSC immunomodulatory properties. Existing data are still inconclusive: one study found impaired immunosuppressive properties in adult AA patients, manifested by deficient in vitro inhibition of T cell proliferation [103], while another study found normal immunosuppressive properties for pediatric AA patients [108]. Nevertheless, recently AA-derived BM-MSCs were shown to have a differential gene expression profile compared to normal MSCs, in many signal pathways including steroid biosynthesis, cell cycle control, adipogenesis-cytokine signaling, adhesion molecules, and TGF-*β* signaling pathway [105], strongly suggesting altered cellular function.

## 5. Chronic Idiopathic Neutropenia

Chronic idiopathic neutropenia (CIN) is a benign disorder of granulopoiesis, characterized by a prolonged reduction of circulating neutrophils, because of increased apoptosis of the granulocytic progenitor cells [109, 110]. CIN is dominated by similar immunopathologic features to AA: presence of activated immune cells, such as T lymphocytes or monocytes and elevated levels of proinflammatory and proapoptotic cytokines in patient bone marrow (such as TNF-*α*, IL-1*β*, IL-6, IFN*γ*, and FasL).

MSCs in CIN patients have a normal BM frequency and normal immunophenotype and can normally differentiate to adipocytes, osteocytes, and chondrocytes. CIN-derived MSCs produce normal levels of TNF-*α*, IL-1*β*, and IL-6 in culture supernatants, suggesting that BM MSCs are not responsible for the elevated levels in patient marrow. CIN-derived MSC immunosuppressive potential did not differ from normal MSCs, in terms of inhibition of mitogen-induced T cell proliferation [111].

However, patient MSCs produced significantly elevated levels of TGF-*β*1, associated with the −509 C/T TGF-*β*1 single nucleotide polymorphism (SNP) genotype. Moreover, CIN-derived MSCs displayed defective growth potential, as evidenced by defective clonogenic capacity, increased doubling time, and decreased proliferation, which could not be attributed to either increased cellular apoptosis or increased TGF-*β*1 production: a TGF-*β*1 neutralizing antibody could not restore the impaired clonogenicity in patient MSCs. We conclude that, although BM MSCs do not seem to exert a significant role in the immune deregulation associated with CIN, they contribute to the inhibitory microenvironment by overproducing TGF-*β*1, at least in CIN patients displaying the −509 C/T SNP [111].

## 6. Conclusion

Summarizing, MDS-derived BM MSCs are rather normal as regards the typical immunophenotype, yet occasionally display phenotypical aberrations particularly regarding cell size and cell shape. They manifest various growth deficiencies and often bear genetic as well as epigenetic aberrations. Their differentiation potential is possibly deranged, and, additionally, MDS-derived BM MSCs display immune function deregulations and may have impaired haemopoietic supportive function, with altered cytokine and chemokine production (Table 3).

In the case of acquired AA, the existing literature, although very small, suggests that AA-derived BM MSCs are immunophenotypically normal but aberrant in cell morphology. Nevertheless, AA-derived MSCs manifest growth deficiencies and display irregularities in their differentiation capacity. Regarding their immune functions, impaired immunosuppressive properties in adult AA patients have been documented, although existing data are very limited. Differential gene expression profile was reported, suggesting altered cellular function (Table 3). Lastly, BM MSCs in CIN patients display normal immunophenotype and normal differentiation potential, but once more display defective growth potential (Table 3).

Overall, MSCs in immune-mediated bone marrow failure syndromes bear some functional deviations, the most prominent being growth deficiencies, which could contribute to disease pathophysiology. Currently it is unknown if these aberrations are inherent MSC characteristics or most likely are secondary defects imposed by the disease marrow environment.

## Figures and Tables

**Table 1:** Major cytokines and chemokines produced by BM MSCs regulating HSCs.

Cytokines	Chemokines
SCF	CCL2
Flt3 ligand	CCL3
TPO	CCL4
SDF-1	CCL5
TGF-*β*	CCL7
LIF	CCL20
IL-1	CCL26
IL-6	CXCL1
IL-7	CXCL2
IL-8	CXCL5
IL-11	CXCL8
IL-12	CXCL10
IL-14	CXCL11
IL-15	CXCL12
GM-CSF	
M-CSF	

**Table 2:** Immunosuppressive functions of BM MSCs on various immune cell types.

Cell types	MSC function
T cells	Inhibition of T lymphocyte proliferation/activation
B cells	Inhibition of B cell proliferation/maturation/antibody secretion
NK cells	Inhibition of NK proliferation/function
DCs	Inhibition of DC maturation/function
Tregs	Promotion of Treg maturation and cell function

**Table 3:** Summary of BM MSC characteristics in MDS, acquired AA, and CIN patients.

	MDS	Acquired AA	CIN
Immunophenotype	Normal	Normal	Normal
Cell morphology	Aberrant	Aberrant	N.D.
Cell growth	Defective	Defective	Defective
Senescence	Increased	N.D.	N.D.
Multipotency	Defective (?)	Defective	Normal
Genomic profile	Frequent genetic aberrations, unrelated to HSC abnormalities	Aberrant gene expression profile	N.D.
Methylation profile	Altered	N.D.	N.D.
Immune properties	Impaired mainly in low-risk MDS	Impaired (?)	Normal
Haemopoietic support capacity	Impaired (?)	N.D.	N.D.

A question mark (?) indicates that existing data are still inconclusive or contradictory, and N.D. indicates the lack of associated data.

## References

[1] Ellis SL, Nilsson SK (2012). The location and cellular composition of the hemopoietic stem cell niche. *Cytotherapy*.

[2] Cook D, Genever P (2013). Regulation of mesenchymal stem cell differentiation. *Advances in Experimental Medicine and Biology*.

[3] Pittenger MF, Mackay AM, Beck SC (1999). Multilineage potential of adult human mesenchymal stem cells. *Science*.

[4] Pontikoglou C, Delorme B, Charbord P (2008). Human bone marrow native mesenchymal stem cells. *Regenerative Medicine*.

[5] Frenette PS, Pinho S, Lucas D (2013). Mesenchymal stem cell: keystone of the hematopoietic stem cell niche and a stepping-stone for regenerative medicine. *Annual Review of Immunology*.

[6] Thanabalasundaram G, Arumalla N, Tailor HD, Khan WS (2012). Regulation of differentiation of mesenchymal stem cells into musculoskeletal cells. *Current Stem Cell Research and Therapy*.

[7] Woodbury D, Schwarz EJ, Prockop DJ (2000). Adult rat and human bone marrow stromal cells differentiate into neurons. *Journal of Neuroscience Research*.

[8] Zhang L, Ye JS, Decot V (2012). Research on stem cells as candidates to be differentiated into hepatocytes. *Bio-Medical Materials and Engineering*.

[9] Friedenstein AJ, Gorskaja UF, Kulagina NN (1976). Fibroblast precursors in normal and irradiated mouse hematopoietic organs. *Experimental Hematology*.

[10] Huang GT, Gronthos S, Shi S (2009). Critical reviews in oral biology & medicine: mesenchymal stem cells derived from dental tissues vs. those from other sources: their biology and role in Regenerative Medicine. *Journal of Dental Research*.

[11] Lin CS, Lin G, Lue TF (2012). Allogeneic and xenogeneic transplantation of adipose-derived stem cells in immunocompetent recipients without immunosuppressants. *Stem Cells and Development*.

[12] Sabatini F, Petecchia L, Tavian M, De Villeroché VJ, Rossi GA, Brouty-Boyé D (2005). Human bronchial fibroblasts exhibit a mesenchymal stem cell phenotype and multilineage differentiating potentialities. *Laboratory Investigation*.

[13] Karahuseyinoglu S, Cinar O, Kilic E (2007). Biology of stem cells in human umbilical cord stroma: in situ and in vitro surveys. *Stem Cells*.

[14] Dominici M, Le Blanc K, Mueller I (2006). Minimal criteria for defining multipotent mesenchymal stromal cells. The International Society for Cellular Therapy position statement. *Cytotherapy*.

[15] Battula VL, Treml S, Bareiss PM (2009). Isolation of functionally distinct mesenchymal stem cell subsets using antibodies against CD56, CD271, and mesenchymal stem cell antigen-1. *Haematologica*.

[16] Hatzfeld A, Eid P, Peiffer I (2007). A sub-population of high proliferative potential-quiescent human mesenchymal stem cells is under the reversible control of interferon *α*/*β*. *Leukemia*.

[17] Sugiyama T, Kohara H, Noda M, Nagasawa T (2006). Maintenance of the hematopoietic stem cell pool by CXCL12-CXCR4 chemokine signaling in bone marrow stromal cell niches. *Immunity*.

[18] Calvi LM, Adams GB, Weibrecht KW (2003). Osteoblastic cells regulate the haematopoietic stem cell niche. *Nature*.

[19] Sacchetti B, Funari A, Michienzi S (2007). Self-renewing osteoprogenitors in bone marrow sinusoids can organize a hematopoietic microenvironment. *Cell*.

[20] Mayack SR, Wagers AJ (2008). Osteolineage niche cells initiate hematopoietic stem cell mobilization. *Blood*.

[21] Chitteti BR, Cheng Y, Poteat B (2010). Impact of interactions of cellular components of the bone marrow microenvironment on hematopoietic stem and progenitor cell function. *Blood*.

[22] Naveiras O, Nardi V, Wenzel PL, Hauschka PV, Fahey F, Daley GQ (2009). Bone-marrow adipocytes as negative regulators of the haematopoietic microenvironment. *Nature*.

[23] Omatsu Y, Sugiyama T, Kohara H (2010). The essential functions of adipo-osteogenic progenitors as the hematopoietic stem and progenitor cell niche. *Immunity*.

[24] Tzeng YS, Li H, Kang YL, Chen W, Cheng W, Lai D (2011). Loss of Cxcl12/Sdf-1 in adult mice decreases the quiescent state of hematopoietic stem/progenitor cells and alters the pattern of hematopoietic regeneration after myelosuppression. *Blood*.

[25] Méndez-Ferrer S, Michurina TV, Ferraro F (2010). Mesenchymal and haematopoietic stem cells form a unique bone marrow niche. *Nature*.

[26] Sharma M, Afrin F, Satija N, Tripathi RP, Gangenahalli GU (2011). Stromal-derived factor-1/CXCR4 signaling: indispensable role in homing and engraftment of hematopoietic stem cells in bone marrow. *Stem Cells and Development*.

[27] Chabanon A, Desterke C, Rodenburger E (2008). A cross-talk between stromal cell-derived factor-1 and transforming growth factor-*β* controls the quiescence/cycling switch of CD34+ progenitors through FoxO3 and mammalian target of rapamycin. *Stem Cells*.

[28] Lataillade JJ, Clay D, Dupuy C (2000). Chemokine SDF-1 enhances circulating CD34+ cell proliferation in synergy with cytokines: possible role in progenitor survival. *Blood*.

[29] Lataillade JJ, Clay D, Bourin P (2002). Stromal cell-derived factor 1 regulates primitive hematopoiesis by suppressing apoptosis and by promoting G0/G1 transition in CD34+ cells: evidence for an autocrine/paracrine mechanism. *Blood*.

[30] Flores-Figueroa E, Varma S, Montgomery K (2012). Distinctive contact between CD34+ hematopoietic progenitors and CXCL12+ CD271+ mesenchymal stromal cells in benign and myelodysplastic bone marrow. *Laboratory Investigation*.

[31] Dazzi F, Ramasamy R, Glennie S, Jones SP, Roberts I (2006). The role of mesenchymal stem cells in haemopoiesis. *Blood Reviews*.

[32] Kemp KC, Hows J, Donaldson C (2005). Bone marrow-derived mesenchymal stem cells. *Leukemia and Lymphoma*.

[33] da Silva Meirelles L, Fontes AM, Covas DT, Caplan AI (2009). Mechanisms involved in the therapeutic properties of mesenchymal stem cells. *Cytokine and Growth Factor Reviews*.

[34] von Bahr L, Sundberg B, Lönnies L (2012). Long-term complications, immunologic effects, and role of passage for outcome in mesenchymal stromal cell therapy. *Biology of Blood and Marrow Transplantation*.

[35] Di Nicola M, Carlo-Stella C, Magni M (2002). Human bone marrow stromal cells suppress T-lymphocyte proliferation induced by cellular or nonspecific mitogenic stimuli. *Blood*.

[36] Asari S, Itakura S, Ferreri K (2009). Mesenchymal stem cells suppress B-cell terminal differentiation. *Experimental Hematology*.

[37] Corcione A, Benvenuto F, Ferretti E (2006). Human mesenchymal stem cells modulate B-cell functions. *Blood*.

[38] Spaggiari GM, Moretta L (2013). Cellular and molecular interactions of mesenchymal stem cells in innate immunity. *Immunology & Cell Biology*.

[39] Luz-Crawford P, Kurte M, Bravo-Alegria J (2013). Mesenchymal stem cells generate a CD4+CD25+Foxp3+ regulatory T cell population during the differentiation process of Th1 and Th17 cells. *Stem Cell Research & Therapy*.

[40] Chabannes D, Hill M, Merieau E (2007). A role for heme oxygenase-1 in the immunosuppressive effect of adult rat and human mesenchymal stem cells. *Blood*.

[41] Jones BJ, Brooke G, Atkinson K, McTaggart SJ (2007). Immunosuppression by placental indoleamine 2,3-dioxygenase: a role for mesenchymal stem cells. *Placenta*.

[42] Sato K, Ozaki K, Oh I (2007). Nitric oxide plays a critical role in suppression of T-cell proliferation by mesenchymal stem cells. *Blood*.

[43] Selmani Z, Naji A, Zidi I (2008). Human leukocyte antigen-G5 secretion by human mesenchymal stem cells is required to suppress T lymphocyte and natural killer function and to induce CD4+ CD25highFOXP3+ regulatory T cells. *Stem Cells*.

[44] Waterman RS, Tomchuck SL, Henkle SL, Betancourt AM (2010). A new mesenchymal stem cell (MSC) paradigm: polarization into a pro-inflammatory MSC1 or an immunosuppressive MSC2 phenotype. *PLoS ONE*.

[45] Chan JL, Tang KC, Patel AP (2006). Antigen-presenting property of mesenchymal stem cells occurs during a narrow window at low levels of interferon-*γ*. *Blood*.

[46] Krampera M, Cosmi L, Angeli R (2006). Role for interferon-*γ* in the immunomodulatory activity of human bone marrow mesenchymal stem cells. *Stem Cells*.

[47] Prasanna SJ, Gopalakrishnan D, Shankar SR, Vasandan AB (2010). Pro-inflammatory cytokines, IFN*γ* and TNF*α*, influence immune properties of human bone marrow and Wharton jelly mesenchymal stem cells differentially. *PLoS ONE*.

[48] Campo E, Swerdlow SH, Harris NL, Pileri S, Stein H, Jaffe ES (2011). The 2008 WHO classification of lymphoid neoplasms and beyond: evolving concepts and practical applications. *Blood*.

[49] Greenberg P, Cox C, LeBeau MM (1997). International scoring system for evaluating prognosis in myelodysplastic syndromes. *Blood*.

[50] Beris P, Georgiou G (2012). Overview of myelodysplastic syndromes. *Seminars in Hematology*.

[51] Khan H, Vale C, Bhagat T (2013). Role of DNA methylation in the pathogenesis and treatment of myelodysplastic syndromes. *Seminars in Hematology*.

[52] Nimer SD (2008). Myelodysplastic syndromes. *Blood*.

[53] Nolte F, Hofmann W (2008). Myelodysplastic syndromes: molecular pathogenesis and genomic changes. *Annals of Hematology*.

[54] Tiu R, Gondek L, O’Keefe C, Maciejewski JP (2007). Clonality of the stem cell compartment during evolution of myelodysplastic syndromes and other bone marrow failure syndromes. *Leukemia*.

[55] Fozza C, Longinotti M (2013). The role of T-cells in the pathogenesis of myelodysplastic syndromes: passengers and drivers. *Leukemia Research*.

[56] Tauro S, Hepburn MD, Peddie CM, Bowen DT, Pippard MJ (2002). Functional disturbance of marrow stromal microenvironment in the myelodysplastic syndromes. *Leukemia*.

[57] Aizawa S, Nakano M, Iwase O (1999). Bone marrow stroma from refractory anemia of myelodysplastic syndrome is defective in its ability to support normal CD34-positive cell proliferation and differentiation in vitro. *Leukemia Research*.

[58] Raaijmakers MH, Mukherjee S, Guo S (2010). Bone progenitor dysfunction induces myelodysplasia and secondary leukaemia. *Nature*.

[59] Walkley CR, Olsen GH, Dworkin S (2007). A microenvironment-induced myeloproliferative syndrome caused by retinoic acid receptor *γ* deficiency. *Cell*.

[60] Flores-Figueroa E, Arana-Trejo RM, Gutiérrez-Espíndola G, Pérez-Cabrera A, Mayani H (2005). Mesenchymal stem cells in myelodysplastic syndromes: phenotypic and cytogenetic characterization. *Leukemia Research*.

[61] Flores-Figueroa E, Montesinos JJ, Flores-Guzmán P (2008). Functional analysis of myelodysplastic syndromes-derived mesenchymal stem cells. *Leukemia Research*.

[62] Klaus M, Stavroulaki E, Kastrinaki M (2010). Reserves, functional, immunoregulatory, and cytogenetic properties of bone marrow mesenchymal stem cells in patients with myelodysplastic syndromes. *Stem Cells and Development*.

[63] Campioni D, Moretti S, Ferrari L, Punturieri M, Castoldi GL, Lanza F (2006). Immunophenotypic heterogeneity of bone marrow-derived mesenchymal stromal cells from patients with hematologic disorders: correlation with bone marrow microenvironment. *Haematologica*.

[64] Campioni D, Rizzo R, Stignani M (2009). A decreased positivity for CD90 on human mesenchymal stromal cells (MSCs) is associated with a loss of immunosuppressive activity by MSCs. *Cytometry B*.

[65] Lopez-Villar O, Garcia JL, Sanchez-Guijo FM (2009). Both expanded and uncultured mesenchymal stem cells from MDS patients are genomically abnormal, showing a specific genetic profile for the 5q- syndrome. *Leukemia*.

[66] Geyh S, Oz S, Cadeddu RP (2013). Insufficient stromal support in MDS results from molecular and functional deficits of mesenchymal stromal cells. *Leukemia*.

[67] Zhao ZG, Xu W, Yu HP (2012). Functional characteristics of mesenchymal stem cells derived from bone marrow of patients with myelodysplastic syndromes. *Cancer Letters*.

[68] Aanei CM, Eloae FZ, Flandrin-Gresta P (2011). Focal adhesion protein abnormalities in myelodysplastic mesenchymal stromal cells. *Experimental Cell Research*.

[69] Yüksel MK, Topçuoğlu P, Kurdal M, Ilhan O (2010). The clonogenic potential of hematopoietic stem cells and mesenchymal stromal cells in various hematologic diseases: a pilot study. *Cytotherapy*.

[70] Varga G, Kiss J, Várkonyi J (2007). Inappropriate Notch activity and limited mesenchymal stem cell plasticity in the bone marrow of patients with myelodysplasia syndromes. *Pathology and Oncology Research*.

[71] Aanei CM, Flandrin P, Eloae FZ (2012). Intrinsic growth deficiencies of mesenchymal stromal cells in myelodysplastic syndromes. *Stem Cells and Development*.

[72] Zhao Z, Wang Z, Li Q (2012). The different immunoregulatory functions of mesenchymal stem cells in patients with low-risk or high-risk myelodysplastic syndromes. *PLoS ONE*.

[73] Marcondes AM, Bair S, Rabinovitch PS, Gooley T, Deeg HJ, Risques R (2009). No telomere shortening in marrow stroma from patients with MDS. *Annals of Hematology*.

[74] Delorme B, Ringe J, Pontikoglou C (2009). Specific lineage-priming of bone marrow mesenchymal stem cells provides the molecular framework for their plasticity. *Stem Cells*.

[75] Ramakrishnan A, Awaya N, Bryant E, Torok-Storb B (2006). The stromal component of the marrow microenvironment is not derived from the malignant clone in MDS. *Blood*.

[76] Soenen-Cornu V, Tourino C, Bonnet M (2005). Mesenchymal cells generated from patients with myelodysplastic syndromes are devoid of chromosomal clonal markers and support short- and long-term hematopoiesis in vitro. *Oncogene*.

[77] Blau O, Baldus CD, Hofmann W (2011). Mesenchymal stromal cells of myelodysplastic syndrome and acute myeloid leukemia patients have distinct genetic abnormalities compared with leukemic blasts. *Blood*.

[78] Blau O, Hofmann W, Baldus CD (2007). Chromosomal aberrations in bone marrow mesenchymal stroma cells from patients with myelodysplastic syndrome and acute myeloblastic leukemia. *Experimental Hematology*.

[79] Han Q, Sun Z, Liu L (2007). Impairment in immuno-modulatory function of Flk1+CD31-CD34- MSCs from MDS-RA patients. *Leukemia Research*.

[80] Wang Z, Tang X, Xu W (2013). The different immunoregulatory functions on dendritic cells between mesenchymal stem cells derived from bone marrow of patients with low-risk or high-risk myelodysplastic syndromes. *PLoS ONE*.

[81] Zhi-Gang Z, Wei-Ming L, Zhi-Chao C, Yong Y, Ping Z (2008). Immunosuppressive properties of mesenchymal stem cells derived from bone marrow of patient with hematological malignant diseases. *Leukemia and Lymphoma*.

[82] Santamaria C, Muntion S, Roson B (2012). Impaired expression of DICER, DROSHA, SBDS and some microRNAs in mesenchymal stromal cells from myelodysplastic syndrome patients. *Haematologica*.

[83] Guinan EC (2011). Diagnosis and management of aplastic anemia. *Hematology*.

[84] Li JP, Zheng CL, Han ZC (2010). Abnormal immunity and stem/progenitor cells in acquired aplastic anemia. *Critical Reviews in Oncology/Hematology*.

[85] Giannakoulas NC, Karakantza M, Theodorou GL (2004). Clinical relevance of balance between type 1 and type 2 immune responses of lymphocyte subpopulations in aplastic anaemia patients. *British Journal of Haematology*.

[86] Kook H, Zeng W, Guibin C, Kirby M, Young NS, Maciejewski JP (2001). Increased cytotoxic T cells with effector phenotype in aplastic anemia and myelodysplasia. *Experimental Hematology*.

[87] Zonghong S, Meifeng T, Huaquan W (2011). Circulating myeloid dendritic cells are increased in individuals with severe aplastic anemia. *International Journal of Hematology*.

[88] Solomou EE, Rezvani K, Mielke S (2007). Deficient CD4+ CD25+ FOXP3+ T regulatory cells in acquired aplastic anemia. *Blood*.

[89] Dubey S, Shukla P, Nityanand S (2005). Expression of interferon-*γ* and tumor necrosis factor-*α* in bone marrow T cells and their levels in bone marrow plasma in patients with aplastic anemia. *Annals of Hematology*.

[90] Sloand E, Kim S, Maciejewski JP, Tisdale J, Follmann D, Young NS (2002). Intracellular interferon-*γ* in circulating and marrow T cells detected by flow cytometry and the response to immunosuppressive therapy in patients with aplastic anemia. *Blood*.

[91] Zeng W, Miyazato A, Chen G, Kajigaya S, Young NS, Maciejewski JP (2006). Interferon-*γ*-induced gene expression in CD34 cells: identification of pathologic cytokine-specific signature profiles. *Blood*.

[92] Zoumbos NC, Gascon P, Djeu JY, Young NS (1985). Interferon is a mediator of hematopoietic suppression in aplastic anemia in vitro and possibly in vivo. *Proceedings of the National Academy of Sciences of the United States of America*.

[93] Rizzo S, Scopes J, Elebute MO, Papadaki HA, Gordon-Smith EC, Gibson FM (2002). Stem cell defect in aplastic anemia: reduced long term culture-initiating cells (LTC-IC) in CD34+ cells isolated from aplastic anemia patient bone marrow. *Hematology Journal*.

[94] Matsui WH, Brodsky RA, Smith BD, Borowitz MJ, Jones RJ (2006). Quantitative analysis of bone marrow CD34 cells in aplastic anemia and hypoplastic myelodysplastic syndromes. *Leukemia*.

[95] Scopes J, Bagnara M, Gordon-Smith EC, Ball SE, Gibson FM (1994). Haemopoietic progenitor cells are reduced in aplastic anaemia. *British Journal of Haematology*.

[96] Timeus F, Crescenzio N, Doria A (2005). Flow cytometric evaluation of circulating CD34+ cell counts and apoptotic rate in children with acquired aplastic anemia and myelodysplasia. *Experimental Hematology*.

[97] Killick SB, Cox CV, Marsh JCW, Gordon-Smith EC, Gibson FM (2000). Mechanisms of bone marrow progenitor cell apoptosis in aplastic anaemia and the effect of anti-thymocyte globulin: examination of the role of the Fas-Fas-L interaction. *British Journal of Haematology*.

[98] Risitano AM, Kook H, Zeng W, Chen G, Young NS, Maciejewski JP (2002). Oligoclonal and polyclonal CD4 and CD8 lymphocytes in aplastic anemia and paroxysmal nocturnal hemoglobinuria measured by V*β* CDR3 spectratyping and flow cytometry. *Blood*.

[99] Zeng W, Maciejewski JP, Chen G, Young NS (2001). Limited heterogeneity of T cell receptor BV usage in aplastic anemia. *Journal of Clinical Investigation*.

[100] Zoumbos NC, Gascon P, Djeu JY (1985). Circulating activated suppressor T lymphocytes in aplastic anemia. *New England Journal of Medicine*.

[101] Luther-Wyrsch A, Nissen C, Wodnar-Filipowicz A (2001). Intracellular Fas ligand is elevated in T lymphocytes in severe aplastic anaemia. *British Journal of Haematology*.

[102] Maciejewski JP, Selleri C, Sato T, Anderson S, Young NS (1995). Increased expression of Fas antigen on bone marrow CD34+ cells of patients with aplastic anaemia. *British Journal of Haematology*.

[103] Bacigalupo A, Valle M, Podestà M (2005). T-cell suppression mediated by mesenchymal stem cells is deficient in patients with severe aplastic anemia. *Experimental Hematology*.

[104] Xu Y, Takahashi Y, Wang Y (2009). Downregulation of GATA-2 and overexpression of adipogenic gene-PPAR*γ* in mesenchymal stem cells from patients with aplastic anemia. *Experimental Hematology*.

[105] Li J, Yang S, Lu S (2012). Differential gene expression profile associated with the abnormality of bone marrow mesenchymal stem cells in aplastic anemia. *PLoS ONE*.

[106] Chao Y, Peng C, Harn H, Chan C, Wu K (2010). Poor potential of proliferation and differentiation in bone marrow mesenchymal stem cells derived from children with severe aplastic anemia. *Annals of Hematology*.

[107] Shipounova IN, Petrova TV, Svinareva DA, Momotuk KS, Mikhailova EA, Drize NI (2009). Alterations in hematopoietic microenvironment in patients with aplastic anemia. *Clinical and Translational Science*.

[108] Xu Y, Takahashi Y, Yoshimi A, Tanaka M, Yagasaki H, Kojima S (2009). Immunosuppressive activity of mesenchymal stem cells is not decreased in children with aplastic anemia. *International Journal of Hematology*.

[109] Papadaki HA, Palmblad J, Eliopoulos GD (2001). Non-immune chronic idiopathic neutropenia of adult: an overview. *European Journal of Haematology*.

[110] Papadaki HA, Eliopoulos AG, Kosteas T (2003). Impaired granulocytopoiesis in patients with chronic idiopathic neutropenia is associated with increased apoptosis of bone marrow myeloid progenitor cells. *Blood*.

[111] Stavroulaki E, Kastrinaki M, Pontikoglou C (2011). Mesenchymal stem cells contribute to the abnormal bone marrow microenvironment in patients with chronic idiopathic neutropenia by overproduction of transforming growth factor-*β*1. *Stem Cells and Development*.

